# The Relationship Between the Utilization of Arterial Blood Gas Analysis and Rehospitalization in Heart Failure: A Retrospective Cohort Study

**DOI:** 10.3389/fcvm.2022.847049

**Published:** 2022-04-26

**Authors:** Xinyu Zhang, Yijun Sun, Hui Zhang, Huixia Lu, Xiaoping Ji

**Affiliations:** The Key Laboratory of Cardiovascular Remodeling and Function Research, The State and Shandong Province Joint Key Laboratory of Translational Cardiovascular Medicine, Department of Cardiology, Cheeloo College of Medicine, Qilu Hospital, Chinese Ministry of Education, Chinese National Health Commission and Chinese Academy of Medical Sciences, Shandong University, Jinan, China

**Keywords:** arterial blood gas analysis, heart failure, PSM, IPTW, rehospitalization

## Abstract

**Background:**

The most common presentation of decompensated HF is dyspnea, and arterial blood gas analysis is an excellent tool for the decision-making process for most dyspneic patients. However, data on the prognostic value of ABG in HF patients are limited. Herein, a retrospective cohort study was conducted to investigate whether the utilization of arterial blood gas analysis was independently associated with re-hospitalization in patients with heart failure.

**Methods:**

As a retrospective cohort study, the relevant clinical data of hospitalized patients admitted to Zigong Fourth People's Hospital, Sichuan, China from December 2016 to June 2019 with a diagnosis of HF were analyzed. The re-hospitalization within 6 months and the use of intravenous diuretic, nitrates, inotropes, or vasopressors were compared between patients with and without arterial blood gas analysis. We used a multivariable logistic regression model, propensity score analysis, and an inverse probability-weighting model to ensure the robustness of our findings.

**Results:**

We included 1,605 patients with heart failure. The overall re-hospitalization rate within 6 months was 38.2%; it was 34.8% and 41.8% for heart failure patients with or without arterial blood gas analysis, respectively. In the inverse probability-weighting model, the use of arterial blood gas analysis was associated with a 26% lower re-hospitalization rate within 6 months.

**Conclusion:**

The performance of arterial blood gas analysis is associated with a 6-month rehospitalization rate benefit in a general population of heart failure patients. This association warrants further investigation.

## Introduction

Many tests and procedures utilized in the treatment of heart failure patients are unverified clinically. When the results are extremely likely to affect patient therapy, an arterial blood gas (ABG) sample should ideally be collected. The need to evaluate the adequacy of patient ventilation, measure the response to therapeutic or diagnostic treatments, monitor the severity and course of a documented disease process, and examine acid-base status are all common indications for an ABG analysis (1). Indications for ABG analysis should, according to the existing literature, be based on the patient's clinical assessment (2). Arterial puncture for ABG analysis, on the other hand, is an invasive operation that might result in artery occlusion, digital embolization, digital ischemia, sepsis, local infection, pseudoaneurysm, hematoma, hemorrhage, and skin necrosis (3).

Routine ABG analysis is not recommended in individuals with heart failure in clinical practice. When a precise measurement of O2 and CO2 partial pressure is required, an ABG analysis should be conducted (i.e., patients with respiratory distress). Patients with cardiogenic shock should have their lactate and pH levels checked. However, there is a lack of evidence on the prognostic significance of ABG in HF patients. The purpose of this study was to investigate if there was an association between ABG analysis and the rehospitalization rate of heart failure patients.

## Participants and Methods

### Study Cohort

The STROBE (Strengthening the Reporting of Observational Studies in Epidemiology) statement was followed in the reporting of this study (4). We retrospectively analyzed the relevant clinical data of hospitalized patients admitted to Zigong Fourth People's Hospital, Sichuan, China from December 2016 to June 2019 with a diagnosis of HF (4, 5). Heart failure was defined according to the European Society of Cardiology (ESC) criteria (2). The study aimed to investigate whether ABG analysis contributes to reductions in re-hospitalization rates and other clinically significant changes in the management of heart failure patients independently. The ethics committee of Zigong Fourth People's Hospital gave its approval to the study (Approval Number: 2020-010). Because of the study's retrospective nature, informed consent was not required. The study adheres to the Helsinki Declaration. The ABG group consisted of patients who had arterial blood gas taken at the first day of admission, whereas the non-ABG group consisted of the rest of the patients. The content of the analysis included pH, standard residual base, standard bicarbonate, partial pressure of carbon dioxide, total carbon dioxide, methemoglobin, haematocrit blood gas, reduced hemoglobin, potassium ion, chloride ion, sodium ion, glucose blood gas, lactate, measured residual base, measured bicarbonate, carboxyhemoglobin, body temperature blood gas, oxygen saturation, partial oxygen pressure, oxyhemoglobin, anion gap, free calcium, and total hemoglobin.

### Primary Outcome and Secondary Outcomes

The primary outcome was rehospitalization within 6 months. Rehospitalization was defined as the first rehospitalization within 6-month time period after discharge from the index hospitalization. Unfortunately, the reason of rehospitalization was not clarifed in this public database. The secondary outcomes included offering intravenous diuretic, nitrates, inotropes, or vasopressors therapy.

### Statistical Analysis

All of the participants were subjected to descriptive analysis. Numbers and percentages were used to represent categorical variables. For normal distributions, continuous variables were reported as mean and standard deviation (SD), and for skewed distributions, median and interquartile range. For categorical, regularly distributed, and non-normally distributed continuous variables, we employed the chi-square test, one-way ANOVA, and Kruskal-Wallis tests, respectively.

Propensity score matching (PSM) (6) was used to minimize the effect of confounding variables like disease severity, which may lead to outcome bias. The propensity score was calculated using a multivariate logistic regression model and was based on the likelihood of a patient receiving blood gas analysis. With a caliper width of 0.02 and a one-to-one closest neighbor matching algorithm, The following variables were used to generate the propensity score: demographic characteristics, body Mass Index (BMI), pulse, mean arterial pressure (MAP), respiratory rate, cardiac function classification, medical history (myocardial infarction, congestive heart failure, peripheral vascular disease, cerebrovascular disease, chronic obstructive pulmonary disease, diabetes, chronic kidney disease, acute renal failure), Charlson comorbidity index (CCI) score, white blood cell count (WBC), red cell distribution width (RDW), platelet count, albumin, sodium, potassium, chloride, blood urea nitrogen (BUN, creatinine, estimated glomerular filtration rate (eGFR), brain natriuretic peptide (BNP), troponin and creatine kinase. The degree of PSM was measured using a standardized mean difference (SMD). A value of <0.1 was deemed acceptable. Finally, 556 matched pairs were generated and used in further studies. An inverse probability of treatment weighting (IPTW) model was utilized to produce a weighted cohort using the calculated propensity scores as weights. The propensity score was then adjusted using univariable logistic regression.

All the analyses were performed with the statistical software packages R (http://www.R-project.org, The R Foundation) and Free Statistics software versions 1.3. A threshold of *p* < 0.05 (two-sided) was considered statistically significant.

## Results

### Population and Baseline Characteristics

**Figure 1:**
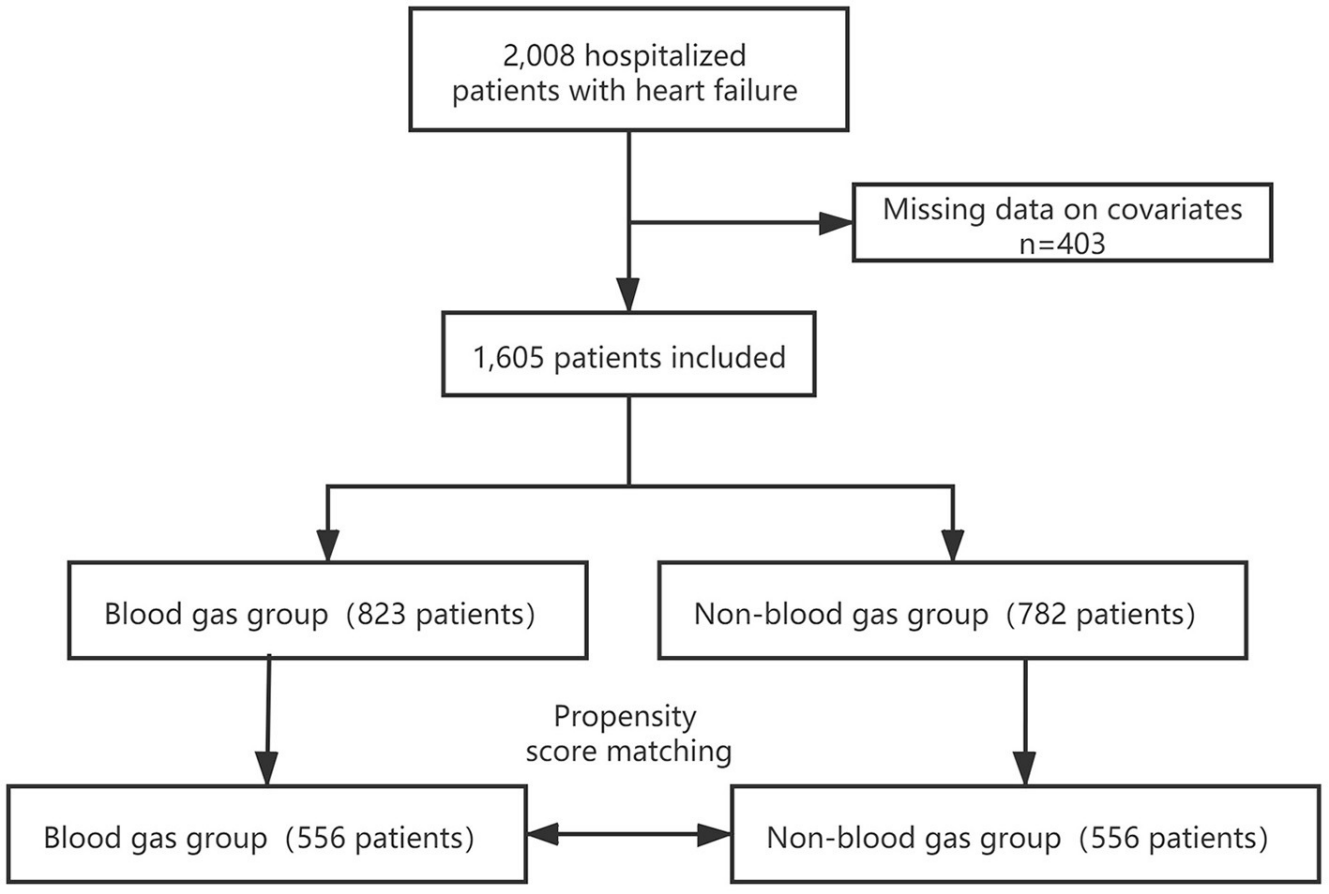
Flow chart of the screening and enrollment of study participants.

We included 1,605 patients in our study cohort (Figure 1). Blood gas was analyzed for 51.3% of patients during their admission. The characteristics of the original cohort are summarized in Table 1. In the original cohort, a larger percentage of the ABG patients were male (45.4%), at an advanced age (41.3%), in NYHA cardiac function classification IV (35.6%) or Killip grade ≥II(79.7%) and had higher Pulse (86.91 ± 21.49), Respiration rate (19.30 ± 1.70), White blood cell (7.71±3.77 × 10^9^/*L*), Troponin (0.43 ± 2.72 pg/ml), BNP (1,392.51 ± 1,462.23 pg/ml), CCI score, consisted of more Peripheral vascular disease (6.4%), Chronic obstructive pulmonary disease (COPD) (16.3%), Diabetes (26.5%) and Chronic kidney disease (25.3%) than no ABG group. The opposite patterns were observed in Albumin and Sodium.

**Table 1:** Baseline characteristics of participants.

**Covariate**	**Original cohort**			**Matched cohort**		
	**No blood gas**	**Blood gas**	**SMD**	**No blood gas**	**Blood gas**	**SMD**
*n*	782	823		556	556	
Age ≥ 80 years old (%)	264 (33.8)	340 (41.3)	0.156	205 (36.9)	204 (36.7)	0.004
Female (%)	479 (61.3)	449 (54.6)	0.136	326 (58.6)	321 (57.7)	0.018
BMI, kg/m^2^ [mean (sd)]	22.04 (16.27)	21.42 (3.93)	0.053	21.38 (6.14)	21.49 (3.93)	0.021
MAP, mmHg [mean (sd)]	94.52 (15.58)	95.80 (16.00)	0.081	95.54 (15.82)	95.65 (16.10)	0.007
Pulse, /min [mean (sd)]	82.95 (20.74)	86.91 (21.49)	0.187	84.23 (20.65)	85.18 (21.14)	0.045
Respiration, /min [mean (sd)]	18.86 (1.63)	19.30 (1.70)	0.263	18.90 (1.43)	19.07 (1.36)	0.117
NYHA cardiac function classification (%)			0.252			0.093
II	148 (18.9)	128 (15.6)		91 (16.4)	102 (18.3)
III	445 (56.9)	402 (48.8)		302 (54.3)	313 (56.3)
IV	189 (24.2)	293 (35.6)		163 (29.3)	141 (25.4)
Killip grade (%)			0.311			0.031
I	255 (32.6)	167 (20.3)		144 (25.9)	139 (25.0)
II	386 (49.4)	435 (52.9)		295 (53.1)	294 (52.9)
III	125 (16.0)	197 (23.9)		103 (18.5)	109 (19.6)
IV	16 (2.0)	24 (2.9)		14 (2.5)	14 (2.5)
Myocardial infarction (%)	46 (5.9)	68 (8.3)	0.093	37 (6.7)	40 (7.2)	0.021
Congestive heart failure (%)	720 (92.1)	764 (92.8)	0.029	516 (92.8)	501 (90.1)	0.097
Peripheral vascular disease (%)	30 (3.8)	53 (6.4)	0.118	28 (5.0)	28 (5.0)	<0.001
Cerebrovascular disease (%)	52 (6.6)	63 (7.7)	0.039	34 (6.1)	38 (6.8)	0.029
Chronic obstructive pulmonary disease (%)	57 (7.3)	134 (16.3)	0.282	52 (9.4)	35 (6.3)	0.114
Diabetes (%)	153 (19.6)	218 (26.5)	0.165	130 (23.4)	120 (21.6)	0.043
Chronic kidney disease (%)	163 (20.8)	208 (25.3)	0.105	125 (22.5)	123 (22.1)	0.009
Acute renal failure (%)	3 (0.4)	3 (0.4)	0.003	0 (0.0)	2 (0.4)	0.085
CCI score (%)			0.357			0.102
0	27 (3.5)	23 (2.8)		16 (2.9)	23 (4.1)
1	347 (44.4)	270 (32.8)		218 (39.2)	231 (41.5)
2	276 (35.3)	282 (34.3)		205 (36.9)	190 (34.2)
3	111 (14.2)	185 (22.5)		97 (17.4)	90 (16.2)
4	17 (2.2)	55 (6.7)		17 (3.1)	20 (3.6)
5	4 (0.5)	7 (0.9)		3 (0.5)	2 (0.4)
6	0 (0.0)	1 (0.1)		0 (0.0)	0 (0.0)
White blood cell, ×10^9^/*L* [mean (sd)]	7.04 (3.23)	7.71 (3.77)	0.189	7.23 (3.38)	7.26 (3.28)	0.011
RDW, % [mean (sd)]	14.91 (2.02)	14.82 (1.90)	0.047	14.85 (1.94)	14.85 (1.94)	<0.001
Platelet, ×10^9^/*L* [mean (sd)]	142.61 (63.50)	146.44 (64.00)	0.06	143.42 (62.75)	146.12 (64.09)	0.042
Albumin, g/L [mean (sd)]	37.05 (4.95)	36.25 (4.86)	0.162	36.66 (4.83)	36.58 (4.91)	0.016
Sodium, mmol/L [mean (sd)]	138.60 (4.38)	138.07 (5.23)	0.109	138.58 (4.45)	138.42 (4.97)	0.034
Potassium, mmol/L [mean (sd)]	3.97 (0.65)	3.99 (0.74)	0.022	3.94 (0.62)	3.97 (0.74)	0.032
Chloride, mmol/L [mean (sd)]	102.17 (5.46)	101.76 (6.41)	0.07	102.34 (5.44)	102.10 (6.07)	0.042
BUN, mmol/L [mean (sd)]	9.54 (5.26)	9.67 (5.77)	0.024	9.41 (5.10)	9.38 (5.75)	0.004
Creatinine, μmol/L [mean (sd)]	110.50 (82.99)	108.80 (75.74)	0.021	105.70 (74.17)	108.15 (78.68)	0.032
eGFR, mL/min/1.73 m2 [mean (sd)]	67.28 (35.11)	69.53 (37.73)	0.061	69.67 (36.22)	69.83 (35.92)	0.004
BNP, pg/ml [mean (sd)]	1,189.64 (1,234.07)	1,392.51 (14,62.23)	0.15	1,259.75 (1,289.32)	1,318.20 (1,420.01)	0.043
Troponin, pg/ml [mean (sd)]	0.19 (1.12)	0.43 (2.72)	0.112	0.22 (1.31)	0.22 (1.50)	0.002
Creatine kinase, IU/L [mean (sd)]	122.23 (137.60)	148.11 (365.72)	0.094	105.70 (74.17)	108.15 (78.68)	0.032

*BMI, body Mass Index; MAP, mean arterial pressure; NYHA, New York heart association; CCI, Charlson Comorbidity Index; BUN, blood urea nitrogen; Egfr, estimated glomerular filtration rate; BNP, brain natriuretic peptide*.

### Primary Outcome

**Figure 2:**
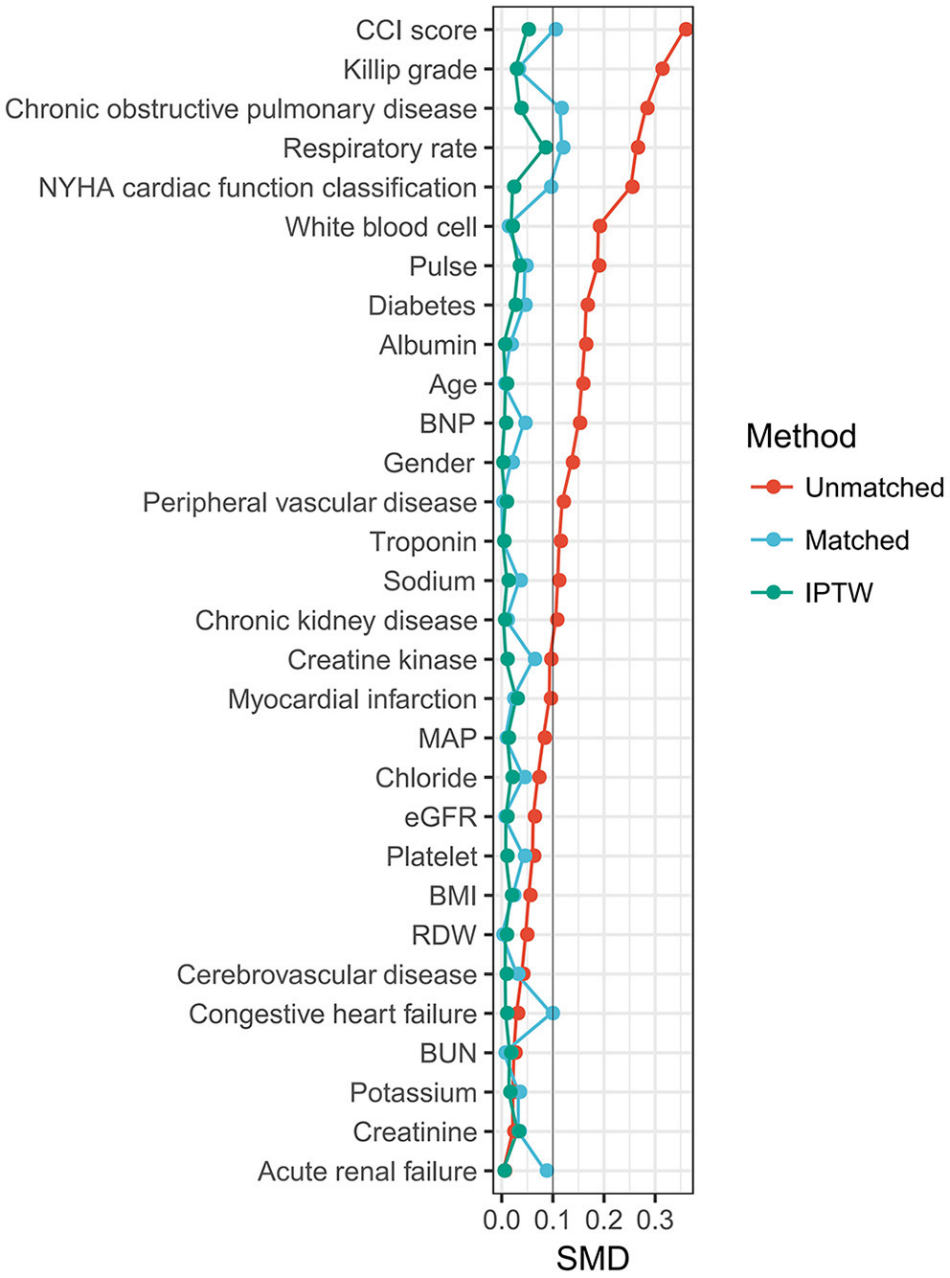
Relative influence factor of covariates. The relative influence factor measures how discriminative the 30 covariates of the propensity score model are when predicting the likelihood of ABG performance.

The 30 variables were initially used to create a propensity score model. Figure 2 illustrates the contributions of individual factors to the final propensity score. CCI score, Killip grade, history of COPD, respiration rate, and NYHA cardiac function classification are among the top variables: unsurprisingly, these covariates influence clinicians' judgments on whether or not to perform ABG analysis. IPW was used to normalize the differences between the ABG and no ABG cohorts based on the estimated propensity scores. Most of the weighted cohort variables were comparable or “balanced” across the groups with and without ABG analysis, as indicated in Table 1. The exceptions were respiration rate, history of COPD, and CCI score. A regression model was developed to adjust for these unbalanced covariates on the weighted cohort.

**Figure 3:**
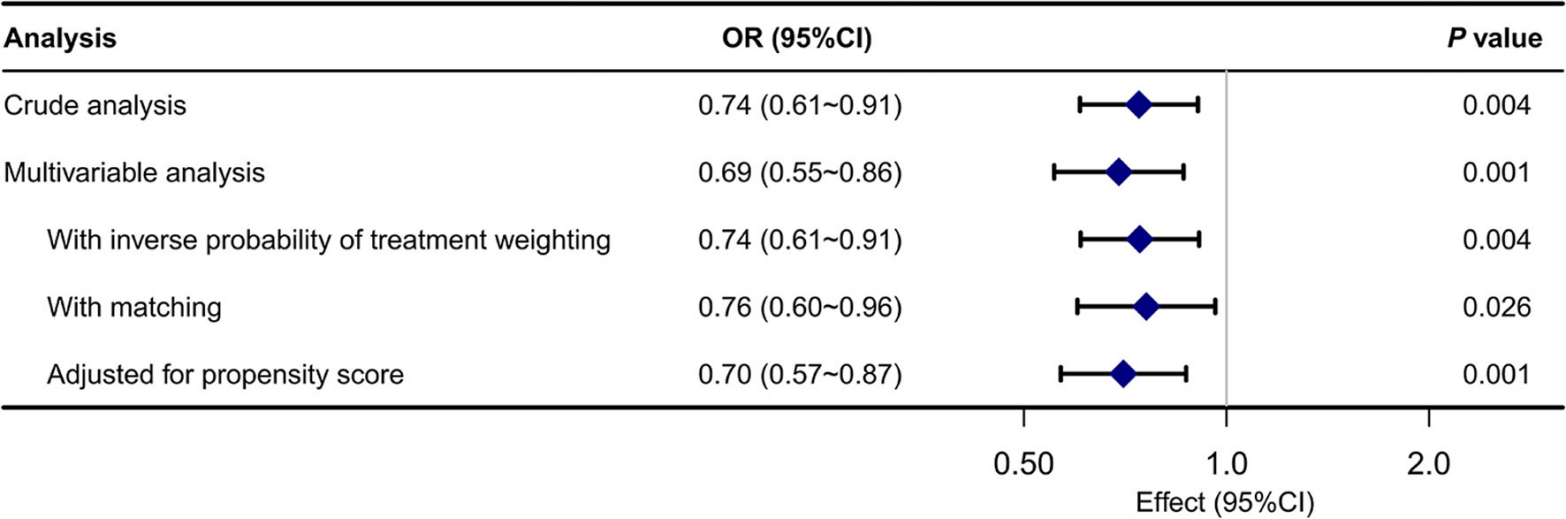
Associations between ABG analysis and the outcome in the crude analysis, multivariable analysis, and propensity-score analyses.

The rehospitalization rates were 41.8% (327/782) and 34.8% (286/ 823) for the no ABG group and ABG group in the original cohort, respectively. IPW demonstrated a significantly lower rehospitalization rate in the ABG group. The OR was 0.74 (95% CI, 0.61–0.91, *P* = 0.004). The propensity score-matched rehospitalization rates for the no ABG group and ABG group were 41.4 and 34.9%, respectively. In multivariable logistics regression, the OR was 0.76 (95% CI, 0.60–0.96, *P* = 0.026) (Figure 3).

### Secondary Outcome Studies With Propensity Score Matching

We evaluated several secondary outcomes to investigate what factors could have contributed to the ABG analysis's advantages following PSM. There were several significant changes in secondary outcomes. In the beginning, ABG patients were considerably more likely to utilize intravenous diuretics (89 vs. 82.7%, *p* = 0.003). Second, ABG patients were significantly more likely to take intravenous nitrates (12.1 vs. 7%, *p* = 0.006). Finally, there was no significant difference in the use of intravenous inotropes or vasopressors in the two groups.

## Discussion

Patients with heart failure who are admitted to the hospital have a poor prognosis. Precise risk stratification might aid in the development of the best treatment options for these patients. Cardiogenic pulmonary edema causes pulmonary edema with impaired gas exchange (hypoxemia, hypercapnia), and insufficient cardiac output causes decreased tissue perfusion, leading to metabolic acidosis in HF patients with cardiogenic pulmonary edema. Thus, in high-risk HF patients, arterial blood gas analysis may be utilized as a comprehensive marker for backward and forward failure, and acid-base balance can be used to determine the general condition of heart function (7). However, there were conflicting opinions on the use of arterial blood gas analysis. According to Blum FE et al., a large reduction in the number of ABGs acquired in the intensive care unit has no detrimental influence on patient outcome or safety, and a reduction in the number of ABGs per patient allows for cost-effective patient care with a decreased risk of complications (1).

Dyspnea is the most prevalent symptom of decompensated HF, and ABG analysis is an excellent tool for most dyspneic patients' decision-making. Their risk classification might be based on the acid-base balance indicated by ABG. However, evidence is scarce on the prognostic significance of ABG in HF patients. In 588 AHF patients (prevalence of acidosis 8.5%), Minana et al. demonstrated that arterial PO_2_, PCO_2_, and pH upon admission were not associated with all-cause long-term mortality, discouraging the use of ABG for prognosis evaluation (8), whereas Alkalosis was associated with increased mortality in 621 AHF patients hospitalized to critical care units, according to Shirakabe et al. (9). Kato et al. demonstrated that the lower the PaCO2 level at admission, the greater the risk of long-term all-cause mortality in AHF patients (10). Meanwhile, according to a study by Park et al. pH has additional predictive value and can be utilized to improve risk classification and management in high-risk AHF patients (7). These findings highlighted the clinical importance of ABG analysis at admission in AHF patients. The significant variances in the study populations may be the cause of the discrepancy. In this propensity score-matched cohort trial, ABG analysis recipients had a lower risk-adjusted rehospitalization rate within 6 months than patients who did not receive ABG analysis. This relationship was shown to be reliable in additional models.

Patients with ABG had a higher severity of illness score, more concomitant illnesses, and a higher BNP level in our study. Despite these indicators indicating a worse group of patients, we observed that after adjusting for confounding, patients who received ABG analysis had a substantially decreased rehospitalization rate after 6 months. There was an obvious selection bias in the study population since the criteria for ABG were not previously specified, and more “critical-looking” individuals were more likely to undergo ABG analysis.

Clinicians may choose to perform ABG tests early in the stay for patients with heart failure, considering the factors shown in Figure 2. We evaluated various factors between patients with and without ABG and tested several different hypotheses to explain the rehospitalization rate benefit. Intravenous diuretics and nitrates were used more often in the group who received ABG analysis. But it is not certain whether the ABG triggered the use of intravenous diuretics and nitrates or if it had already been in place. Whether the rehospitalization rate improvements are entirely due to the differences in intravenous diuretics and nitrates use is impossible to assess given the sample size. Our findings suggest that ABG may offer physicians information that might help them in the management of heart failure patients. The initial ABG may be used to identify individuals who are at the highest risk and require more invasive and intense therapy. We understand that observational database studies like this need meticulous, diverse, and rigorous statistical methodologies to deliver meaningful, trustworthy, and actionable findings. We believe that we have done so for the subject at hand, and plan to pursue further such analyses in the future to reduce the ambiguity of clinical decision-making in a complicated and complex context.

The findings should not be interpreted as the final and conclusive word on the value of ABG in the management of heart failure. The potential issues of residual confounding by variables not captured in the electronic health record, as well as the generalizability of the findings to other institutions, require further investigation as an observational single-center study retrospectively performed on electronic health record data. Because practice patterns may have changed over the study period, the outcomes were not adjusted for the year of admission, which is a limitation of the analysis. For several analyses, prospective randomized trials will be required for confirmation.

## Conclusion

In a broad population of heart failure patients, ABG analysis performance is associated with a 6-month rehospitalization rate benefit. The cause of this advantage is unknown; however, it might be related to the increasing use of intravenous diuretics and nitrates as suggested by ABG values. This relationship needs further investigation.

## Data Availability Statement

Publicly available datasets were analyzed in this study. This data can be found here: https://doi.org/10.13026/8a9e-w734.

## Ethics Statement

The studies involving human participants were reviewed and approved by the Ethics Committee of Zigong Fourth People's Hospital. Written informed consent for participation was not required for this study in accordance with the national legislation and the institutional requirements.

## Author Contributions

XZ: methodology and writing. YS and HZ: data curation and validation. HL and XJ: writing—review and editing. All authors contributed to the article and approved the submitted version.

## Funding

This work was supported by the National Natural Science Foundation of China (81900444, 81873516, 81873522, and 82170463), the Clinical Research Center of Shandong University (2020SDUCRCA009), the National Key Research and Development Program of China (2017YFC1308303 and 2021YFF0501403), Z-2019-42-1908-2 and Z-2016-23-2101 project of China International Medical Foundation, and the Natural Science Foundation of Shandong Province (ZR2019PH030, ZR2020QH007, and ZR2019BH052). The funders had no role in the study design, data collection, and analysis, decision to publish, or preparation of the manuscript.

## Conflict of Interest

The authors declare that the research was conducted in the absence of any commercial or financial relationships that could be construed as a potential conflict of interest.

## Publisher's Note

All claims expressed in this article are solely those of the authors and do not necessarily represent those of their affiliated organizations, or those of the publisher, the editors and the reviewers. Any product that may be evaluated in this article, or claim that may be made by its manufacturer, is not guaranteed or endorsed by the publisher.
